# Toward clinically actionable AI: ensuring accountability and equitable implementation in oral cancer screening

**DOI:** 10.1097/JS9.0000000000002943

**Published:** 2025-07-02

**Authors:** Rui Shi, Ling Gao, Jing-Jing Zheng

**Affiliations:** a Department of Oral and Maxillofacial Reconstruction, The Affiliated Hospital of Qingdao University, Qingdao, China; b School of Stomatology, The Qingdao University, Qingdao, China; c Department of Oral and Maxillofacial Surgery, The Affiliated Hospital of Qingdao University, Qingdao, China; d Key Lab of Oral Clinical Medicine, The Affiliated Hospital of Qingdao University, Qingdao, Shandong, China; e Department of Endodontics, The Affiliated Hospital of Qingdao University, Qingdao, China

*Dear Editor,*

We commend the authors on their systematic analysis in the recent review article “Diagnostic accuracy of artificial intelligence assisted clinical imaging in the detection of oral potentially malignant disorders and oral cancer: a systematic review and meta-analysis^[1,2]^.” Building upon this work and incorporating our clinical experience with OSCC cases, we wish to supplement these findings with the following perspectives:

Point 1: While the study confirms the excellent diagnostic performance of AI-assisted clinical photography (DOR = 77.772, sensitivity 93.9%), its implementation in resource-limited settings faces practical challenges: Strong dependence on image quality: In primary care settings, non-specialist photography is vulnerable to interference from lighting conditions, angles, and mucosal reflections. This may lead to feature loss or misclassification—for example, glare may be misinterpreted as pathological leukoplakia, while subtle textural changes of early invasive carcinoma may be undetectable in low-resolution images. Limited case coverage: The analysis predominantly included typical lesions, whereas real-world screening must address complex presentations (e.g., visual similarity between oral lichen planus and premalignant lesions). An unpublished community study in India showed AI sensitivity for atypical lesions plummeting to 78%, indicating limited model generalizability. We therefore propose developing embedded smartphone AI for real-time quality control (e.g., automated detection of blurring or uneven illumination) and conducting multicenter validation trials in primary care with geographically diverse cases to enhance model robustness.

Point 2: Multimodal technology integration is key to overcoming single-modality limitations^[3]^. We recommend establishing a tiered screening framework—primary screening via clinical photography at grassroots levels, with positive cases referred to regional centers for OCT verification (e.g., using Hong Kong’s EfficientNet-B0 + PSOCT algorithm). Concurrently, developing multimodal fusion models that combine macroscopic features from clinical photography with OCT-based stromal layer analysis could significantly improve invasive carcinoma detection rates (preliminary animal studies show AUC increasing from 0.87 to 0.93).

Point 3: Algorithmic transparency and accountability frameworks are fundamental to clinical implementation. Current deep convolutional network models lack interpretability for lesion assessment (e.g., unclear heatmap justification for misclassifying traumatic ulcers as cancerous), potentially leading to overreliance on AI and delayed biopsies. Ambiguous liability boundaries between developers, operators, and institutions when AI false-negative results cause diagnostic delays further complicate implementation. The EU Medical AI Accountability Directive (2023) now mandates traceable decision logs. To address this technically, mandate integrated explanation modules (e.g., Grad-CAM heatmaps highlighting suspicious regions); Procedurally, establish human-AI collaborative protocols—AI-positive screenings require clinician confirmation, while negative results warrant periodic follow-ups considering risk factors (smoking/alcohol history); Policy-wise, urge WHO to develop an ethical framework for oral AI, explicitly assigning developers responsibility for algorithm generalization validation.

This study provides crucial evidence for AI-driven oral cancer screening, but clinical translation requires real-world validation (Point 1), technological integration (Point 2), and ethical governance (Point 3). Future work should prioritize developing low-cost OCT devices and cross-population model validation.

## Data Availability

Not applicable.
